# Hypothesis: inflammatory acid-base disruption underpins Long Covid

**DOI:** 10.3389/fimmu.2023.1150105

**Published:** 2023-04-14

**Authors:** Vicky van der Togt, Jeremy S. Rossman

**Affiliations:** ^1^ Research-Aid Networks, Chicago, IL, United States; ^2^ School of Biosciences, University of Kent, Canterbury, United Kingdom

**Keywords:** Long Covid, PASC, COVID-19, SARS-CoV-2, inflammation, acid-base, acidosis

## Abstract

The mechanism of Long Covid (Post-Acute Sequelae of COVID-19; PASC) is currently unknown, with no validated diagnostics or therapeutics. SARS-CoV-2 can cause disseminated infections that result in multi-system tissue damage, dysregulated inflammation, and cellular metabolic disruptions. The tissue damage and inflammation has been shown to impair microvascular circulation, resulting in hypoxia, which coupled with virally-induced metabolic reprogramming, increases cellular anaerobic respiration. Both acute and PASC patients show systemic dysregulation of multiple markers of the acid-base balance. Based on these data, we hypothesize that the shift to anaerobic respiration causes an acid-base disruption that can affect every organ system and underpins the symptoms of PASC. This hypothesis can be tested by longitudinally evaluating acid-base markers in PASC patients and controls over the course of a month. If our hypothesis is correct, this could have significant implications for our understanding of PASC and our ability to develop effective diagnostic and therapeutic approaches.

## Introduction

It is estimated that 15% of people infected with SARS-CoV-2 have long-term post-acute symptoms, known as PASC or ‘Long Covid’ (1), with higher rates seen in women, hospitalized individuals and those with underlying metabolic disorders (1–3). To qualify as PASC, symptoms must last a minimum of two months, and often include a wide range of variable symptoms. PASC can affect all ages and can occur after even mild infections. The cause of PASC is currently debated but likely involves several different components, including: viral dissemination and persistence, immune activation and dysregulation (e.g. clotting, autoantibodies and reactivation of pre-existing latent viruses), cell death and tissue damage, and long-lasting cellular changes (e.g. epigenetic changes, senescence, fibroproliferation, metabolism alterations) (3). However, at present we lack an integrative understanding of how these disease components interact to cause the variable symptomology of PASC. Based on studies on acute COVID-19, PASC and the related myalgic encephalomyelitis (ME; chronic fatigue syndrome), we hypothesize that an inflammatory acid-base disruption underpins PASC and that viral proteins, both acutely and persistently-expressed, cause disease symptomology through disseminated tissue damage and inflammatory acid-base disruptions.

## Inflammatory acid-base disruptions in PASC

In PASC, inflammation reduces microvascular blood flow (e.g. through endothelial inflammation, platelet and erythrocyte aggregation, clotting and neutrophil extracellular trap formation) (3), creating a hypoxic environment that causes cellular metabolic changes (e.g. anaerobic respiration) and altered tissue and immune functions (4). SARS-CoV-2 proteins also directly cause metabolic changes (5) similar to hypoxia, increasing anaerobic respiration and the generation of lactate and protons. Significant or persistent production of protons can exceed the cellular and systemic buffering capacity, causing localized or systemic acidosis that results in a range of symptoms, including muscle fatigue similar to that experienced after strenuous anaerobic exercise. As SARS-CoV-2 vRNA and proteins have been found in muscle tissue (6), this shift to anaerobic respiration may cause acidosis in skeletal, cardiac and smooth muscle even in the absence of strenuous exercise, leading to the most common symptoms of PASC: fatigue and muscle weakness (3). In PASC patients, abnormally high blood lactate has been found after even mild exertion, suggesting metabolic dysfunction and muscle acidosis (7). In ME, muscle usage also results in intramuscular acidosis with increased acid clearance time (8), suggesting that post-exertional malaise may be caused by persistent muscle acidosis following repeated use of hypoxic and metabolically-reprogrammed muscle tissue.

However, the body compensates for acidosis in multiple ways: by increasing the elimination of acidic compounds in the urine, by increasing bicarbonate production in the kidneys, by altering the expression of lactate dehydrogenase genes (LDH; mediating the interconversion of pyruvate to lactate) and by altering respiration to modulate the levels of CO_2_, and thus carbonic acid in the blood (9). In PASC patients, hyperventilation (10) may reflect a compensatory response to acidosis, lowering carbonic acid in the blood. However, over-compensation can lead to alkalosis, which is also seen in acute SARS-CoV-2 infections. 73% of patients with moderate-to-severe COVID-19 present with either acidosis or alkalosis (11), with acidosis or compensated respiratory alkalosis significantly increasing the risk of death (12). Similarly, acute disease outcomes were worse in patients with high or low blood bicarbonate levels (13) and in those with elevated LDH (14), suggesting that acidosis may play a role in the pathogenesis of acute COVID-19 (15). Additionally, dehydration during the acute infection, which can impair clearance of excess acid or base, increases the likelihood of developing PASC (16).

The effects of acid-base imbalance can affect any tissue, generating many of the symptoms of PASC, including brain fog, though acidosis in the blood does not typically affect the brain as the blood-brain barrier (BBB) is not freely permeable to protons. However, SARS-CoV-2 and viral proteins increase BBB permeability (17), which may enable the flow of protons into the brain. In addition, viral proteins have been found in the brain (3, 17) and may mediate metabolic reprogramming, inflammation and hypoxia. The resulting anaerobic respiration may directly affect the acid-base balance in the brain, as indicated by elevated lactate levels in the cerebrospinal fluid (CSF) in ME patients (18). Acidosis has been shown to impair executive functions (19), as seen in PASC patients suffering from brain fog (3). Altered acid-base balance can also have a myriad of additional effects, as protons may directly act as neurotransmitters (20) and inflammation-inducing damage-associated molecular patterns (21). Altered brain acidity affects multiple neurological conditions, such as anxiety, possibly through inhibitory action (22) on the TRPM3 ion channel (23), the activity of which is reduced in PASC patients (24), and through activation of acid-sensing ion channels (25). In addition, extracellular acidosis can disrupt the cellular molecular clock (26), potentially affecting circadian rhythms and causing the sleep disturbances often reported by PASC patients (3). Sleep is critical for glymphatic clearance (27) of metabolic and toxic substances from the brain and its inhibition may further disrupt the acid-base balance and cause a build-up of toxins (e.g. amyloid-β fibrils) that could eventually lead to neurodegenerative disease.

Together, the variable tissue damage, dysregulated inflammation and acid-base disruptions caused by persistent SARS-CoV-2 infection or proteins can cause the range of symptoms seen in PASC (**Table 1**).

**Table 1:** PASC symptoms and proposed disease pathways.

Organ System	Symptoms	Disease Pathways
Systemic	Fatigue, PEM, temperature changes (fever / chills)	IN, DI, AC
Circulatory system	Hypoxia and reduced blood flow, clotting, bradycardia, tachycardia, fainting, chest pain, visible veins, covid toes	TD, BF, IN, AC
Respiratory system	Shortness of breath, cough, sore throat, hyperventilation / altered breathing, sneezing, sinus pain / congestion, low oxygen saturation	TD, BF, IN, AC
Nervous system	POTS, dizziness, coordination / balance issues, tremors, loss of sensation, loss/changes of smell / taste, hearing issues, vision issues, headaches, insomnia	TD, BF, IN, DI, AC
Nervous system (cognitive)	Brain fog, confusion, attention issues, memory loss, speech / language issues, emotional/mood issues	TD, BF, IN, DI, AC
Musculoskeletal system	Muscle pain, muscle fatigue / tightness, bone / joint pain / swelling	TD, BF, IN, DI AC
Immune and lymphatic systems	Inflammation, new allergies, anaphylaxis	IN, DI, AC
Gastrointestinal system	Constipation, diarrhoea, nausea, vomiting, acid reflux, abdominal pain, change in appetite, changes in weight	TD, BF, IN, AC, microbiome alterations
Integumentary system	Rashes, hair loss, peeling skin, changes in sweat	TD, BF, IN, AC
Endocrine, reproductive, urinary systems	Menstrual changes, bladder issues, hormone changes / issues	TD, BF, IN, AC

TD, tissue damage; BF, blood flow; IN, inflammation; DI, dysfunctional immunity (including viral reactivation and autoimmunity); AC, acidosis (including response mechanisms). Symptoms based on (28). An individual’s symptom profile will likely be shaped by differences in the amount of persistent virus/proteins, the extent of viral dissemination and tissue damage, previous infections, existing metabolic or inflammatory diseases and the impact of daily activities and circadian / hormonal cycles.

## Hypothesis testing

We propose that acid-base disruptions underpin PASC disease. This hypothesis can be tested by examining acid-base markers and proxies (**Table 2**) in three groups of diverse participants: a) patients with active, medically-diagnosed untreated PASC disease, b) participants with a confirmed COVID-19 test that are at least two-months post-infection without on-going symptoms, c) participants that have never tested positive, have no on-going symptoms and are serologically-negative for antibodies against the SARS-CoV-2 N protein (avoiding miss-classification due to vaccination with the Spike protein). It should be noted that at this point in the pandemic, with over 680 million cases worldwide, waning antibody levels, the presence of asymptomatic infections and, in some areas, limited access to testing, group (c) will likely include some participants that have been previously infected. This is likely impossible to avoid, may somewhat reduce the effect size and will need to be accounted for in the analysis and interpretation. All participants should also be matched on demographic factors (i.e. age, sex, socioeconomic factors), pre-existing co-morbidities and vaccination status (including, manufacturer, doses and timing), as these factors can influence immunity, metabolism and PASC disease course (1–3, 29–31).

**Table 2:** Evaluating acid-base imbalances in PASC patients.

Tests	Evaluation Method
Exam and cognitive assessment 6-MWT with ABG and lactate testing	Upon arrival at the clinic participants should be seated for 30 min in a quiet room before a physical examination and preparation of an indwelling arterial catheter. A blood sample is taken for full ABG measurement (including lactate, bicarbonate, and anion gap) before insertion of an inter-arterial sensor for continuous pH and blood-gas monitoring (e.g., Paratrend 7). Participants then remain seated for a further 30min while taking the Montreal cognitive assessment before beginning the 6-MWT, with earlobe capillary lactate measurements every minute. After the test, participants would rest, reclined for 30min before the catheter and sensor are removed.
Home blood lactate Home smO_2_ Symptom reporting	Participants would be provided with a home blood lactate meter (e.g., Abbott Lingo wearable for continuous use or the Edge capillary home lactate meter for repeated manual measurements), a wearable smO_2_ meter (e.g., Moxy sensor) and a wearable activity monitor (e.g., smartwatch). Data would be recorded continually during the day (both at rest and during any activities) with intermittent overnight monitoring. Participants would use a log to track their daily symptoms, activity levels and sleep, noting any changes in symptoms/intensity as they occur.

Blood pH, bicarbonate, CO_2,_ and lactate can then be assessed through arterial blood gas (ABG) measurements before, during and after a 6-minute walk test (6-MWT). This data can be correlated with a physical examination, cognitive assessment, and self-reported symptoms. It is expected that PASC patients would show variably altered acid-base parameters at rest. During the 6-MWT, PASC patients would be expected to show increased lactate, decreased pH and compensating decreases in CO_2_ compared to control participants. It is possible that some healthy, previously-infected, participants would also show altered parameters due to residual impact of the infection. If any results indicate a blood pH < 7.1 or lactate ≥ 5nmol/l then additional diagnostic exams and treatment for acute acidosis may be needed during the testing (9). As PASC may be a type of chronic metabolic acidosis, a single treatment (e.g. bicarbonate) is unlikely to offer full disease resolution, but any changes in symptomology following treatment would further support the hypothesis.

However, a single exam may be insufficient to detect an acid-base imbalance as PASC symptoms vary over time and are affected by activity levels and circadian/hormonal cycles (e.g. blood lactate levels for a set exertion are higher in women during the mid-follicular menstrual phase) (32). To account for this variability, the above diagnostic tests can be supplemented with one month of continual home monitoring using muscle oxygen saturation (smO_2_) and blood lactate monitors as proxies for muscle hypoxia and blood pH levels. This data can then be correlated with smartwatch activity monitoring and self-reported symptom surveys. It is expected that PASC patients would show variable but significantly decreased smO_2_ and elevated lactate at rest compared to controls, with further deviations occurring with exertion, affecting symptomology. From these diagnostic and home tests it should be possible to determine if PASC patients have an acid-base imbalance and if this is correlated with activity and symptomology. This would significantly affect our understanding of PASC and offer the possibility of minimally-invasive PASC diagnostic assays (e.g., capillary blood lactate levels on exertion).

## Discussion

We propose the testable hypothesis that PASC is underpinned by an inflammatory acid-base disruption. If the proposed evaluations confirm the hypothesis, this offers both a diagnostic pathway for PASC and suggestions for treatment. As PASC may be a reinforcing cycle of tissue damage, blood flow and oxygenation impairment, dysregulated inflammation, and acid-base disruption, then a treatment protocol could be designed to simultaneously address each disease component through pharmaceutical and/or non-pharmaceutical interventions. It is possible that PASC is not the only pathogen-induced inflammatory acid-base disorder, as several other pathogens cause persistent disease. ME, in particular, shares many features with PASC and the symptom profile of both diseases overlaps with that of acute and chronic acidosis (9, 33). Thus, the proposed hypothesis may be of broader relevance. Additionally, it is possible that even people without persistent symptoms following SARS-CoV-2 infection harbor residual tissue damage and viral proteins, increasing their risk of new health conditions in the future (34). There are still many unanswered questions and the data from this hypothesis testing is urgently needed.

## Data availability statement

The original contributions presented in the study are included in the article/supplementary material. Further inquiries can be directed to the corresponding authors.

## Author contributions

VT conceived of the hypothesis, VT and JR refined the hypothesis, JR wrote and VT and JR edited the manuscript. All authors contributed to the article and approved the submitted version.
